# Compounds from *Olea europaea* and *Pistacia lentiscus* inhibit oral microbial growth

**DOI:** 10.1186/s12906-019-2461-4

**Published:** 2019-02-26

**Authors:** Lamprini Karygianni, Manuel Cecere, Aikaterini Argyropoulou, Elmar Hellwig, Alexios Leandros Skaltsounis, Annette Wittmer, Jörg Philipp Tchorz, Ali Al-Ahmad

**Affiliations:** 1 Clinic for Preventive Dentistry, Periodontology and Cariology, Center of Dental Medicine, University of Zurich, Zürich, Switzerland; 2 Department of Operative Dentistry and Periodontology, Medical Center, Faculty of Medicine, University of Freiburg, Freiburg, Germany; 3 Department of Pharmacognosy and Natural Products Chemistry, Faculty of Pharmacy, National and Kapodistrian University of Athens, Athens, Greece; 4 PharmaGnose S.A., Papathanasiou 24, 34100 Chalkida, Euboea Greece; 5 Institute of Medical Microbiology and Hygiene, Faculty of Medicine, University of Freiburg, Freiburg, Germany; 6 Department of Operative Dentistry, Periodontology and Endodontology, University of Dental Medicine and Oral Health, Danube Private University (DPU), Krems, Austria

**Keywords:** Natural compounds, *Olea europaea*, *Pistacia lentiscus*, Oral microorganisms, Minimum inhibitory concentration (MIC), Minimum bactericidal concentration (MBC)

## Abstract

**Background:**

In view of the increasing antibiotic resistance, the introduction of natural anti-infective agents has brought a new era in the treatment of bacterially derived oral diseases.

**Methods:**

The aim of this study was to investigate the antimicrobial potential of five natural constituents of *Olea europaea* (oleuropein, maslinic acid, hydroxytyrosol, oleocanthal, oleacein) and three compounds of *Pistacia lentiscus* (24Z-isomasticadienolic acid, oleanolic acid, oleanonic aldehyde) against ten representative oral bacterial species and a *Candida albicans* strain. After the isolation and quality control of natural compounds, the minimum inhibitory concentration (MIC) and the minimum bactericidal concentration (MBC) assay were performed.

**Results:**

Among all *O. europaea*-derived constituents, maslinic acid was the most active (MIC = 4.9–312 μg mL^− 1^, MBC = 9.8–25 μg mL^− 1^) one against oral streptococci and anaerobic pathogenic bacteria (*Porphyromonas gingivalis*, *Fusobacterium nucleatum*, *Parvimonas micra*), while oleuropein, hydroxytyrosol, oleocanthal and oleacein showed milder, yet significant effects against *P. gingivalis* and *F. nucleatum*. Among all *P. lentiscus* compounds, oleanolic acid was the most effective one against almost all microorganisms with MIC values ranging from 9.8 μg mL^− 1^ (*P. gingivalis*) to 625 μg mL^− 1^ (*F. nucleatum*, *P. micra*). In the presence of 24Z-isomasticadienolic acid, a mean inhibitory concentration range of 2.4 μg mL^− 1^ to 625 μg mL^− 1^ was observed for strict anaerobia. The MIC value for 24Z-isomasticadienolic acid was estimated between 39 μg mL^− 1^ (*Streptococcus sobrinus*, *Streptococcus oralis*) and 78 μg mL^− 1^ (*Streptococcus mutans*). All tested compounds showed no effects against *Prevotella intermedia.*

**Conclusions:**

Overall, maslinic acid and oleanolic acid exerted the most significant inhibitory activity against the tested oral pathogens, especially streptococci and anaerobic oral microorganisms.

## Background

The nature-inspired therapies of various bacterially-driven infections based on herbals are one of the most current therapeutic trends in medicine [1]. Nevertheless, more than 300.000 plant extracts need to be screened for their antioxidant and antimicrobial properties [2–4]. Among these, a selection of Mediterranean plant extracts from olive (*Olea europaea*), parsley (*Petroselinum crispum*), oregano (*Origanum vulgare*), thyme (*Thymus vulgaris*), sage (*Salvia officinalis*), mastic gum (*Pistacia lentiscus*) and false yellowhead (*Inula viscosa*) have shown a significant inhibitory activity against numerous bacteria [5–7]. However, to elucidate the mechanisms relating to their complex biological behavior, the effect of pure plant-derived compounds on microorganisms has to be investigated [8, 9].

The antimicrobial traits of plants can be attributed to the activity of natural antibiotics with low molecular weight (MW < 500), named phytoalexins, and synergistic action [10, 11]. These well-studied antimicrobial stress-derived metabolites of plant origin include flavonoids, glycosteroids, terpenoids, and polyphenols [12]. Furthermore, other specific defense mechanisms of plants are supported by the release of avirulence (Avr) gene-activated resistance (R) proteins or the secretion of a polysaccharide with (1–3)-ß-D-glucan subunits, namely callose, under the threat of the microbial invaders [13, 14]. Finally, plants produce endogenous antimicrobial peptides with less than 100 amino acid residues, low acquisition of resistance and broad-spectrum antimicrobial features [15].

Surprisingly, trillions of microbes are an integral part of the healthy human body and outnumber host cells by 10 to 1 [16]. Although microbes could benefit humans by carrying 8 million health-related genes, they are also able to turn into pathogenic body inhabitants under specific circumstances [17, 18]. The high pathogenicity of bacteria, viruses, and fungi can be demonstrated through the formation of antibiotic-resistant oral biofilms [19]. The oral cavity is a representative ecological niche with more than 700 microbial residents, often organized in biofilm networks on teeth or gingiva [20]. As a result, biofilm-associated oral diseases such as caries, gingivitis or periodontitis can occur [21].

In recent years the research on the chemotherapeutic intransigence of microbial biofilms, whose antibiotic resistance is 1000 times higher compared to planktonic bacterial cells, has been intensified [22]. In the oral cavity in particular, the antibiotic-resistant *Enterococcus faecalis* detected in infected root canals expressed the endocarditis-related antigen A (*EfaA*) [23]. Due to the production of β-lactamases by *Prevotella* spp.*,* fusobacteria and capnocytophaga, an abundance of ^*bla*^*TEM* resistance genes could be identified in subgingival and tongue samples [24]. Therefore, there is an urgent need for novel oral antimicrobials with low risk of provoking bacterial resistance to antibiotic monotherapy [3]. In that context, the scenario of introducing novel phytopharmaceuticals has attracted attention lately [25, 26]. The effectiveness of natural antimicrobial candidates can be attributed to their synergistic impact and broad pharmaceutical spectrum resulting from secondary metabolic reactions [27, 28].

For this purpose, the present report focused on the antimicrobial behavior of natural compounds deriving from *Olea europaea L.* (Oleaceae) and *Pistacia lentiscus L.* (Anacardiaceae) against representative oral bacterial species. More specifically, eight different antimicrobial agents from olive leaves, table olive processing wastewater, olive oil and mastic gum were screened against eight representative bacterial inhabitants of the oral cavity, namely *Streptococcus mutans*, *Streptococcus sobrinus*, *Streptococcus oralis*, *Enterococcus faecalis*, *Porphyromonas gingivalis*, *Parvimonas micra*, *Prevotella intermedia*, *Fusobacterium nucleatum* and the yeast *Candida albicans*. Among these *S. mutans* and *S. sobrinus* are related to dental caries [29], *E. faecalis* correlates with secondary endodontic infections [30], *P. gingivalis*, *P. micra* and *P. intermedia* are periodontal pathogens [31]*, while C. albicans* can cause oral infections in denture wearers [32].Typical representatives of the intestinal and skin flora such as *Escherichia coli* and *Staphylococcus aureus*, respectively, were used as reference bacteria. In our previous report, an olive extract and total mastic extract from *P. lentiscus* exhibited significant antimicrobial activity against oral microorganisms [33]. The null hypothesis of this report was that the tested natural compounds from *O. europaea* and *P. lentiscus* have no antimicrobial effect on oral microbes. To assess this, two antimicrobial assays - the minimum bactericidal concentration (MBC) and the minimum inhibitory concentration (MIC) assay were applied.

## Methods

Following our research on the antimicrobial efficacy of Mediterranean natural plant extracts, some of the most promising extracts were selected [33] and their major compounds were tested.

### Compounds extraction from *O. europaea*

Oleuropein and maslinic acid used in this study were isolated from an extract deriving from *O. europaea* L. (Oleaceae) leaves that were collected in 2009 at the region of Attica and were identified by Dr. E. Kalpoutzakis. A voucher specimen is deposited at the herbarium of the Department of Pharmacognosy and Natural Products Chemistry, Faculty of Pharmacy, University of Athens, Greece under the number PROK 006. The preparation of the extract has been described in a previous report [33]. The extract (360 g) was separated, dried and subjected to medium pressure Liquid Chromatography (MPLC) with silica (Si) gel 60 Merck (15–40 mm), using the dichloromethane (CH_2_Cl_2_) / methanol (MeOH) gradient as the eluent to extract pure oleuropein (≥ 95%) and maslinic acid (≥ 95%). Hydroxytyrosol was isolated from an extract produced from table olive processing wastewater. The preparation of the extract and the isolation of pure hydroxytyrosol (≥ 95%) have been previously described [34]. For the procurement of oleacein and oleocanthal the total polyphenol fraction (TPF) of extra virgin olive oil (EVOO) was used as starting material [35], while for their isolation column chromatography (CC) and preparative Thin Layer Chromatography (TLC) were employed, as previously described [36]. Briefly, 170 L of EVOO was subjected to 25 kg of the adsorbent XAD-7HP resin. The resin was activated with water (H_2_O) and ethanol (EtOH), and EVOO remained in the XAD-7HP resin for 2 days with controlled and smoothed shaking and filtered. The resin was washed with 30 L cyclohexane (cHex) for the removal of the lipophilic constituents, and then the polyphenol-enriched extract was obtained by the extraction of the resin with approx. 40 L of ethanol. Further purification was achieved by liquid–liquid extraction using cHex and EtOH, and the obtained ethanolic fraction was filtered through paper and evaporated until dry affording 150 g of TPF. In continuation, 250 mg of TPF were subjected to a Si gel (0.015–0.04 mm) column (25 × 2.7 cm) and mixtures of CH_2_Cl_2_ and MeOH in increasing polarity (0–10% MeOH) were used for the elution. From the fractions obtained using 98:2 CH_2_Cl_2_ / MeOH, oleocanthal was isolated, while from the fractions obtained using 97:3 CH_2_Cl_2_ / MeOH, oleacein was attained. For further purification, preparative TLC was used. Specifically, precoated TLC silica 60 F254 plates, 2 mm layer thickness (purchased from Aldrich), were used while 94:6 CH_2_Cl_2_ / MeOH was used as the mobile phase. Spots were visualized using ultraviolet (UV) light and vanillin-sulfuric acid reagent. Finally, oleocanthal (≥ 95%) and oleacein (≥ 95%) were purified.

### Compounds extraction from *P. lentiscus*

24Z-isomasticadienolic acid, oleanolic acid and oleanonic aldehyde were isolated from mastic gum [37]. Commercially available mastic gum was supplied by Chios Mastic Growers Association which is the exclusive worldwide producer of the resin. Conventional extraction of mastic gum for the preparation of total mastic extract without polymer (TMEWP) (extraction A) has been described in a previous report (Karygianni et al., 2014a). TMEWP partitioned between aqueous 5% Na_2_CO_3_ and ether as already described (extraction B) [38]. The organic phase was reextracted three times with 5% Na_2_CO_3_ (extraction C) and afforded the neutral fraction of mastic (135 g) as the organic phase. The aqueous phase was added to that of extraction B and acidified with 1 N HCl. The acidic solution was reextracted with ether (extraction D), and the organic phase afforded the acid fraction of mastic (190 g).

The acidic fraction (20 g) was submitted to MPLC over normal-phase silica gel first with a cHex / CH_2_Cl_2_ gradient and then with a CH_2_Cl_2_/MeOH gradient affording 23 fractions. In continuation, oleanolic acid was separated by MPLC over normal-phase silica gel eluted with a CH_2_Cl_2_ / MeOH gradient, while 24Z-isomasticadienolic acid by column chromatography over silica gel eluted with a CH_2_Cl_2_ / MeOH gradient. A part of the neutral fraction (17.2 g) was submitted to column liquid chromatography over normal-phase silica gel with a cHex / CH_2_Cl_2_ gradient to afford 22 fractions. Oleanonic aldehyde was separated in continuation by MPLC over normal-phase silica gel eluted with a cHex / CH_2_Cl_2_ gradient.

### Chemical analysis of compounds

**Fig. 1:**
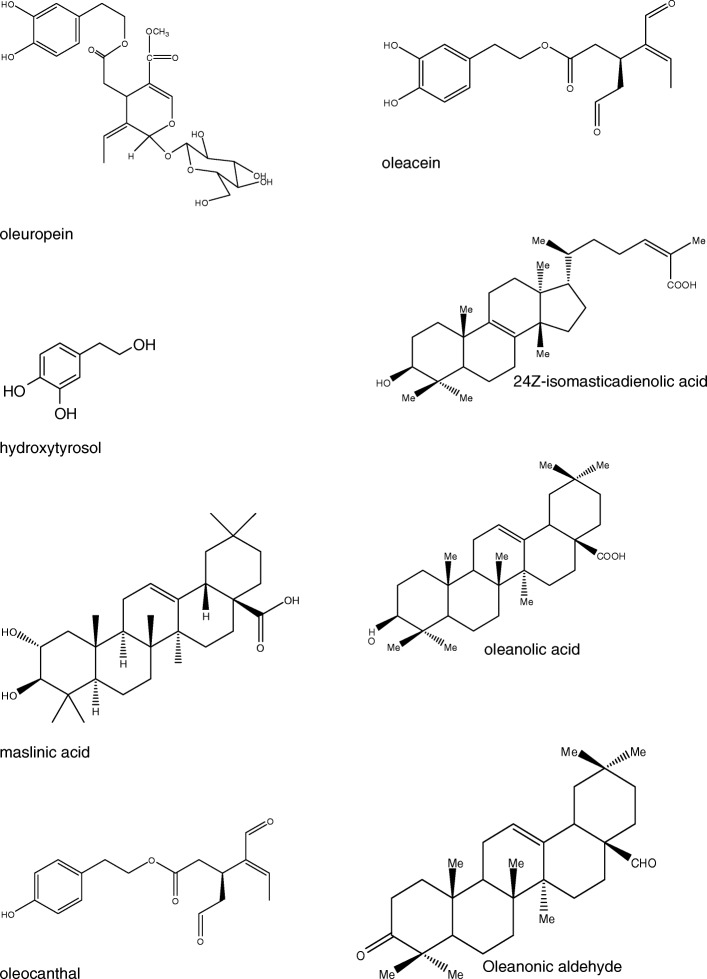
Chemical structures of the tested compounds from *Olea europaea* (oleuropein, maslinic acid, hydroxytyrosol, oleocanthal, oleacein) and *Pistacia lentiscus* (24Z-isomasticadienolic acid, oleanolic acid, oleanonic aldehyde)

The chemical structures of the tested compounds are demonstrated on Fig. 1. The identity and purity (≥95%) of the isolated compounds were confirmed by nuclear magnetic resonance spectroscopy (NMR), mass spectrometry (MS) and high performance liquid chromatography (HPLC) experiments and by comparison with literature data. All solvents (ethanol, methanol, dichloromethane and cyclohexane) were of p.A. quality and came from Merck (Darmstadt, Germany). Water was purified by double distillation.

### Bacterial strains and *Candida albicans*

A total of eleven microbial strains from ten different bacterial strains and one *Candida albicans* strain were tested. Among these eight bacterial strains and *C. albicans* represent typical oral inhabitants, while the reference bacterial strains *Escherichia coli* and *Staphylococcus aureus* are members of the intestinal and skin flora, respectively. Their use facilitated the comparison of the antimicrobial activity of the natural compounds within the oral cavity against their general inhibitory impact. Facultative anaerobic Gram-positive species such as *Streptococcus mutans* DSM 20523, *Streptococcus sobrinus* DSM 20381, *Streptococcus oralis* ATCC 35037, *Enterococcus faecalis* ATCC 29212 and *S. aureus* ATCC 25923 were tested. *Escherichia coli* ATCC 25922 served also as a facultative anaerobic bacterium but with a Gram-negative cell wall. The tested obligate anaerobes included *Porphyromonas gingivalis* W381, *Prevotella intermedia* ATCC 25611, *Fusobacterium nucleatum* ATCC 25586 and *Parvimonas micra* ATCC 23195. All bacterial strains and *C. albicans* were kindly provided by the Division of Infectious Diseases and the Institute of Medical Microbiology and Hygiene of the Albert-Ludwigs-University, Freiburg. The microorganisms were kept at − 80 °C in basic growth medium containing 15% (*v*/v) glycerol prior to use.

### Determination of the minimum inhibitory concentration (MIC)

As described in the Clinical and Laboratory Standards Institute (CLSI) guidelines an overnight culture of each bacterial strain and *C. albicans* was prepared and each dilution was placed on Columbia blood agar plates (CBA, Becton Dickinson GmbH, Heidelberg, Germany) or yeast-cysteine blood agar plates (HCB, Becton Dickinson GmbH, Heidelberg, Germany) [39, 40]. CBA agar plates were used for the incubation of facultative anaerobic bacteria and *C. albicans* at 37 °C and 5 -10% CO_2_ atmosphere for 24 h. HCB agar plates were used for the incubation of anaerobic bacteria at 37 °C for 48 h (anaerobic chamber, Genbox BioMérieux SA, Marcy/Etoile, France). For the microdilution assay at 10^6^ colony forming units (CFU) mL^− 1^ for each strain, Mueller-Hinton Broth (MHB) was utilized for the inoculation of all facultative anaerobic strains, Wilkins–Chalgren broth (WCB) for anaerobic bacteria and Sabouraud Dextrose Broth (SDB) for *C. albicans*. Then, with the aid of a multi-channel pipette appropriate volumes of the MHB/WCB/SDB microbial cultures were transferred into a 96-well microtiter-plate. Each well of the 96-well microtiter-plate had a total volume of 200 μL. Afterwards the prepared compounds were dissolved in dimethyl sulfoxide (DMSO, Sigma, Steinheim, Germany) and diluted in distilled water. A concentration series ranging from 2500 μg mL-^1^ to 2.4 μg mL^− 1^ at dilution levels starting from 2-fold to 512-fold was used to screen all compound solutions in DMSO. The experiments were conducted in duplicate. For bacteria and fungi, a 0.5/1A McFarland standard suspension was diluted in normal saline. A dilution series of DMSO was tested in parallel in order to exclude potential inhibitory effects of the DMSO residuals. In case of bacterial growth in the co-tested DMSO dilution series the inhibitory impact of DMSO was taken into account. Wells containing only sterile MHB/WCB/SDB to minimize the possibility of contamination or 0.2% chlorhexidine (CHX) served as positive and negative controls, respectively. Thereafter, facultative anaerobic bacteria and *C. albicans* were incubated at 37 °C and 5 -10% CO_2_ atmosphere for 24 h, while anaerobic bacteria were kept at 37 °C for 48 h (anaerobic chamber, Genbox BioMérieux SA, Marcy / Etoile, France). All assays for each bacterial strain and *C. albicans* were conducted in duplicate. If the MIC values of a specific strain were not identical, the highest minimum inhibitory concentration (MIC) values were taken into account. MIC was determined as the lowest concentration of each compound at which visible inhibition of bacterial growth occurred, and was expressed by the percentage of bacterial growth at that particular concentration.

### Determination of the minimum bactericidal concentration (MBC)

As described in the CLSI guidelines the minimum bactericidal concentration (MBC) could also be determined [39, 40]. After the MIC testing, 10 μL from each well of the 96-well microtiter-plates containing the tested compound concentration series were incubated on agar plates. In particular, Columbia blood agar (CBA) agar plates were used for facultative anaerobic bacteria and *C. albicans* at 37 °C and 5 -10% CO_2_ atmosphere for 2 days. Strictly anaerobic bacteria were incubated on yeast-cysteine blood agar (HCB) plates at 37 °C for 5 days (anaerobic chamber, Genbox BioMérieux SA, Marcy/Etoile, France). The colony forming units (CFU) were determined visually. The MBC was determined as the concentration at which a three-log decrease in bacterial growth (= 99.9%) was detected compared to the positive control. In the presence of variations within the yielded MIC/MBC values after the repetition of the experiments, the highest MIC/MBC values were listed to eliminate false positive results.

## Results

### *O. europaea*

Five compounds (oleuropein, maslinic acid, hydroxytyrosol, oleocanthal and, oleacein) isolated from *O. europaea* by-products (leaves, table olives processing wastewater) and products (olive oil) were screened. Table 1 demonstrates the mean MIC and MBC values for each of the aforementioned *O. europaea* compounds as well as the tested bacterial and fungal strains.

**Table 1:** Antimicrobial activity in μg mL^-1^ of compounds from *O. europaea*

*O. europaea*												
Sample (% dilution in DMSO)	oleuropein (1.8%)		hydroxytyrosol (1.25%)		oleocanthal (2.1%)		oleacein (1.1%)		maslinic acid (6.6%)		DMSO in %	
c / μg mL^−1^	MIC^a^	MBC^b^	MIC	MBC	MIC	MBC	MIC	MBC	MIC	MBC	MIC	MBC
*Streptococcus mutans* DSM 20523	625	625	312	312	1250	1250	1250	1250	19.5	156	6.00	25.00
*Streptococcus sobrinus* DSM 20381	625	1250	625	1250	312	1250	1250	1250	19.5	19.5	10.00	15.00
*Streptococcus oralis* ATCC 35037	1250	1250	1250	1250	1250	1250	1250	1250	19.5	19.5	10.00	10.00
*Enterococcus faecalis* ATCC 29212	1250	1250	1250	2500	1250	1250	1250	1250	39	312	15.00	25.00
*Candida albicans* DSM 1386	1250	1250	1250	2500	625	1250	1250	1250	1250	1250	8.00	8.00
*Escherichia coli* ATCC 25922	1250	1250	1250	2500	1250	1250	1250	1250	1250	1250	10.00	10.00
*Staphylococcus aureus* ATCC 25923	625	625	312	312	1250	1250	625	625	78	625	10.00	25.00
*Porphyromonas gingivalis* W381	625	625	156	312	156	312	312	625	4.9	9.8	12.5	12.5
*Prevotella intermedia* ATCC 25611	NA	NA	312(5d)	NA	NA	NA	625 (5d) NA		NA	NA	3.12 (5d)	3.12
*Fusobacterium nucleatum* ATCC 25586	625	312	312	312	312	312	156	156	312	25	6.25	6.25
*Parvimonas micra* ATCC 23195	1250	1250	1250	1250	312	312	1250	1250	9.8	9.8	6.25 (5d)	12.5

*NA* No activity observed: MIC or MBC were measured at 2500 μg mL^−1^, 5d = values yielded after 5 days of incubation, ^a^MIC = extract concentration at which the optical density (OD) measurement revealed minimal bacterial growth, ^b^MBC = extract concentration at which a three log reduction (99.9%) of the bacterial growth was induced

Overall, maslinic acid was more effective than oleuropein, hydroxytyrosol, oleocanthal and oleacein. Maslinic acid was active against almost all anaerobic bacterial strains, with a mean concentration range of 4.9 μg mL^− 1^ (*Porphyromonas gingivalis*) to 312 μg mL^− 1^ (*Fusobacterium nucleatum*). The obligate anaerobe *Parvimonas micra* (9.8 μg mL^− 1^) were efficiently inhibited, whereas maslinic acid showed no inhibitory effect against *Prevotella intermedia*. For streptococci (*Streptococcus mutans*, *Streptococcus sobrinus*, *Streptococcus oralis*) the MIC value for maslinic acid was estimated at 19.5 μg mL^− 1^, for *Enterococcus faecalis* at 39 μg mL^− 1^, for *Staphylococcous aureus* at 78 μg mL^− 1^. The highest MIC value at 1.25 mg mL^− 1^ was detected for *Escherichia coli* and *Candida albicans*. For obligate anaerobes, maslinic acid showed low MBC values, which ranged from 9.8 μg mL^− 1^ (*P gingivalis*, *P. micra*) to 25 μg mL^− 1^ (*F. nucleatum*). Streptococci such as *S. sobrinus* and *S. oralis* (19.5 μg mL^− 1^) as well as *S. mutans* (156 μg mL^− 1^) were more easily eradicated when compared to facultative anaerobic *E. faecalis* (312 μg mL^− 1^) and *S. aureus* (625 μg mL^− 1^). The highest MBC value at 1.25 mg mL^− 1^ was detected for *E. coli* and *C. albicans*, while *P. intermedia* was not affected at all by maslinic acid.

Oleacein exhibited a milder inhibitory activity against oral microorganisms. The lowest MIC values of oleacein were observed for obligate anaerobes and were between 156 μg mL^− 1^ (*F. nucleatum*) and 1.25 mg mL^− 1^ (*P. micra*). The anaerobic *P. gingivalis* showed also a satisfactory MIC value of 312 μg mL^− 1^ and *P. intermedia* could be inhibited only by oleacein (625 μg mL^− 1^) after 5 days of culture. All other bacterial strains (streptococci, *E. faecalis*, *E. coli*) and *C. albicans* had MIC and MBC values of 1.25 mg mL^− 1^. The MBC values of oleacein for obligate anaerobia were substantially lower ranging from 156 μg mL^− 1^ (*F. nucleatum*), 625 μg mL^− 1^ (*P. gingivalis*) to 1.25 mg mL^− 1^ (*P. micra*).

Oleocanthal also showed an inhibitory effect on oral bacteria. The lowest MIC values of oleocanthal were detected for *S. sobrinus* (312 μg mL^− 1^) as well as obligate anaerobes and varied between 156 μg mL^− 1^ (*P. gingivalis*) and 312 μg mL^− 1^ (*F. nucleatum*, *P. micra*). *C. albicans* was eradicated at 625 μg mL^− 1^, whereas the streptococci and reference strains had MIC and MBC values of 1.25 mg mL^− 1^. Obligate anaerobes (*P. gingivalis*, *F. nucleatum*, *P. micra*) showed the lowest MBC value (312 μg mL^− 1^), while *P. intermedia* did not respond to the treatment with oleocanthal.

Concerning hydroxytyrosol, the lowest compound concentrations of 156 μg mL^− 1^ (*P. gingivalis*), 312 μg mL^− 1^ after 5 days of culture (*P. intermedia*, *F. nucleatum*) exerted bactericidal effect mainly on strict anaerobic, Gram-negative bacteria. From the streptococci, *S. mutans* and *S. sobrinus* presented also satisfactory inhibitory values of 312 μg mL^− 1^ and 625 μg mL^− 1^, respectively. The highest MIC value of hydroxytyrosol (1.25 mg mL^− 1^) was observed for the reference strains, *C. albicans*. *E. faecalis* and *P. micra*. The lowest MBC value of hydroxytyrosol was estimated at 312 μg mL^− 1^ (*P. gingivalis*, *F. nucleatum*, *S. mutans*), while 99.9% of *E. faecalis, S. aureus* and *C. albicans* were eradicated by 2.5 mg mL^− 1^ of hydroxytyrosol.

Finally, oleuropein had the mildest antimicrobial impact on the oral pathogens. The MIC and MBC values of the eradicated microbial strains for oleuropein were between 625 μg mL^− 1^ (*S. mutans, S. aureus, P. gingivalis*) to 1.25 mg mL^− 1^ (*S. oralis. E. faecalis, E. coli, P. micra, C. albicans)*. The lowest MBC value of oleuropein (312 μg mL^− 1^) was observed for *F. nucleatum,* whereas *P. intermedia* was not inhibited by this compound.

### *P. lentiscus*

Table 2 summarizes the MIC and MBC values of the three compounds (24Z-isomasticadienolic acid, oleanolic acid, and oleanonic aldehyde) isolated from *P. lentiscus* for all screened microbial strains.

**Table 2:** Antimicrobial activity in μg mL^-1^ of compounds from *P. lentiscus*

*P. lentiscus*								
Sample (% dilution in DMSO)	24Z-isomasticadienolic acid (3.9%)		oleanolic acid (2.6%)		oleanonic aldehyde (2.4%)		DMSO (in %)	
c / μg mL^− 1^	MIC^a^	MBC^b^	MIC	MBC	MIC	MBC	MIC	MBC
*Streptococcus mutans* DSM 20523	78	156	19.5	39	1250	1250	6.00	25.00
*Streptococcus sobrinus* DSM 20381	39	1250	19.5	39	1250	1250	10.00	15.00
*Streptococcus oralis* ATCC 35037	39	78	19.5	78	1250	1250	10.00	10.00
*Enterococcus faecalis* ATCC 29212	156	1250	78	312	1250	1250	15.00	25.00
*Candida albicans* DSM 1386	1250	1250	1250	1250	1250	1250	8.00	8.00
*Escherichia coli* ATCC 25922	1250	1250	1250	1250	1250	1250	10.00	10.00
*Staphylococcus aureus* ATCC 25923	1250	1250	78	1250	1250	1250	10.00	25.00
*Porphyromonas gingivalis* W381	2.4	9.8	9.8	9.8	625	1250	12.50	12.50
*Prevotella intermedia* ATCC 25611	NA	NA	NA	NA	NA	NA	3.12 (5d)	3.12
*Fusobacterium nucleatum* ATCC 25586	625	625	625	625	1250	1250	6.25	6.25
*Parvimonas micra* ATCC 23195	2.4	9.8	625	1250	1250	1250	6.25 (5d)	12.50

*NA* No activity observed: MIC or MBC were measured at 1250 μg mL^−1^, 5d = values yielded after 5 days of incubation, ^a^MIC = extract concentration at which the optical density (OD) measurement revealed minimal bacterial growth, ^b^MBC = extract concentration at which a three log reduction (99.9%) of the bacterial growth was induced

Among all mastic gum compounds, oleanolic acid was the most effective against almost all microorganisms with MIC values ranging from 9.8 μg mL^− 1^ (*P. gingivalis*) to 625 μg mL^− 1^ (*F. nucleatum*, *P. micra*) for obligate anaerobes. The MIC value for maslinic acid was estimated at 19.5 μg mL^− 1^ for streptococci (*S. mutans*, *S. sobrinus*, *S. oralis*), at 78 μg mL^− 1^ for *E. faecalis* and *S. aureus*. The highest MIC and MBC value of oleanolic acid (1.25 mg mL^− 1^) was detected for *E. coli* and *C. albicans*, whereas *P. intermedia* was not affected at all by oleanolic acid. The mean MBC values for strict anaerobic bacteria were 9.8 μg mL^− 1^ (*P. gingivalis*), 625 μg mL^− 1^ (*F. nucleatum*) and 1.25 mg mL^− 1^ (*P. micra*), whereas higher MBC values were estimated for streptococci at 39 μg mL^− 1^ (*S. mutans*, *S. sobrinus*) and 78 μg mL^− 1^ (*S. oralis*).

Another compound, the 24Z-isomasticadienolic acid also presented a substantial antimicrobial effect against the screened microorganisms. In its presence, a mean inhibitory concentration range of 2.4 μg mL^− 1^ (*P. gingivalis*, *P. micra*) to 625 μg mL^− 1^ (*F. nucleatum*) was observed for strict anaerobia. The MIC value for 24Z-isomasticadienolic acid was estimated between 39 μg mL^− 1^ (*S. sobrinus*, *S. oralis*) and 78 μg mL^− 1^ (*S. mutans*) for streptococci, while *E. faecalis* had a MIC value of 156 μg mL^− 1^. The highest MIC and MBC value of 24Z-isomasticadienolic acid (1.25 mg mL^− 1^) was detected for *E. coli, S. aureus* and *C. albicans*, whereas *P. intermedia* did not respond to the treatment. The lowest MBC value (9.8 μg mL^− 1^) were determined for the obligate anaerobia *P. gingivalis* and *P. micra*, while 78 μg mL^− 1^ and 156 μg mL^− 1^ of the compound killed 99.9% of *S. oralis* and *P. micra* and *S. mutans*, respectively.

Oleanonic aldehyde presented the lowest antimicrobial activity compared to the other two mastic gum compounds. The lowest MIC value of 625 μg mL^− 1^ was found for *P. gingivalis,* whereas all other tested bacterial and fungal strains presented MIC and MBC value of 1.25 mg mL^− 1^. Oleanonic aldehyde proved to be ineffective against *P. intermedia*.

## Discussion

The present report introduced and screened eight antimicrobial compounds originating from *O. europaea* and *P. lentiscus* against nine representative oral pathogens. The efficacy of three different extracts from the aforementioned plants against oral microorganisms was highlighted in a previous own study [33]. To the best of our knowledge, this is the first study on the inhibition of oral microbial growth induced by the antimicrobial agents of *O. europaea* and *P. lentiscus.*

In this study, maslinic acid isolated from leaves of *O. europaea* proved to be highly effective, even in very low concentrations in the range of 9.8–25 μg mL^− 1^, against oral streptococci and anaerobic pathogenic bacteria such as *Porphyromonas gingivalis*, *Fusobacterium nucleatum* and *Parvimonas micra.* These results confirm the findings of a previous report, which also provided evidence of the high antimicrobial potential of maslinic acid (MIC = 15–30 μg mL^− 1^; MBC = 25–50 μg mL^− 1^) against *S. aureus*, *E. coli*, *E. faecalis* and *Pseudomonas aeruginosa* [41]. Maslinic acid belongs to natural pentacyclic triterpenoids, which are able to damage the cell envelope of both Gram-positive and Gram-negative bacteria [42]. Furthermore, carbon-associated R stereochemistry within this organic compound and the production of synthetic maslinic acid derivatives with the presence of sulfur and chlorine atoms and extra hydroxyl group seem to enhance its antimicrobial capacity [41]. In another report, maslinic acid demonstrated improved antibacterial effects (0.9 μg mL^− 1^) compared to the antibiotic kanamycin (0.9 μg mL^− 1^) toward the Gram-positive *Bacillus thuringiensis* and a substantial inhibitory activity against the Gram-negative *E. coli*, *Salmonella enterica* and *Shigella dysenteria* [43]. Nevertheless, maslinic acid failed to eradicate Gram-negative bacteria such as *E. coli*, *P. aeruginosa* and *Klebsiella pneumoniae* in an earlier study [44]. In addition to its inhibitory effect, maslinic acid has proven antioxidant, antitumor and antidiabetogenic activity [45].

The other three compounds isolated from *O. europaea*, namely oleuropein, oleocanthal, hydroxytyrosol and oleacein presented more moderate inhibitory effects compared to maslinic acid against the Gram-negative anaerobic *P. gingivalis* and *F. nucleatum*. Oleuropein is a oleosidic ester of 3,4-dihydroxyphenylethanol [46] isolated from olive leaves. Indeed*,* there are many reports on the high-level antibacterial activity of oleuropein using various microorganisms [47–49]. Recently*,* Bisignano et al. highlighted also the antibacterial action of an oleuropein derivative, namely 3,4-DHPEA-EA, against Gram-positive ATCC strains, food and clinical isolates of *Staphylococcus epidermidis* and *S. aureus* [50]. One possible mechanism of action involves the prevention of the activity repression of lactoperoxidase mediated by hydrogen peroxide (H_2_O_2_). As a result, the increased release of its oxidation product named hypothiocyanite (^−^OSCN), which can penetrate microbial biofilms, leads to enhanced bacteriostatic features [51, 52]. Interestingly, to strengthen its antibacterial behavior lactic acid bacteria such as *Lactobacillus plantarum* are able to hydrolyze and subsequently convert oleuropein into hydroxytyrosol [53]. The phenolic compound hydroxytyrosol was most effective against Gram-negative anaerobic bacteria. This fact is of high importance in oral infections, since Gram negative bacteria such as *P. gingivalis* are associated with periodontal disease while Gram positive microorganisms correlate with periodontal health [54]. In a previous study, 4-hydroxytyrosol also exerted bactericidal activity against the Gram-positive *S. aureus* as well as the virulent staphylococcal enterotoxin A [55]. The antibacterial behavior of hydroxytyrosol (400 μg mL^− 1^) and the combination hydroxytyrosol/gallic acid against *E. coli*, *Klebsiella pneumoniae*, *Streptococcus pyogenes* and *S. aureus* was confirmed in another recent report [56].

Oleocanthal and oleacein constitute two aldehydic compounds of olive oil with great structural similarity [57]. To date, both substances have proven to be natural non-steroidal, antioxidant and anti-inflammatory compounds [58, 59]. Scotece et al. elucidated the active anti-inflammatory role of oleocanthal [60]. In particular, oleocanthal interferes with the activity of lipopolysaccharide (LPS)-stimulated macrophages and chondrocytes hindering nitric oxide (NO), interleukin (IL-6, IL-1β) and tumor necrosis factor α (TNF-α) production [60]. Oleacein was documented to protect the cardiovascular system by decreasing the progression of atherosclerosis and repairing angiotensin II-affected endothelial progenitor cells [59, 61]. With regard to its antibacterial traits, the present study is the first report on the moderate, yet effective inhibitory activity of oleocanthal and oleacein, especially against anaerobic oral pathogens. However, oleanonic aldehyde as well as the other tested extracts proved to be ineffective against the anerobic *P. intermedia*.

Among all tested triterpenoid acids, oleanolic acid was the most active natural pentacyclic triterpenoid (MIC, MBC = 9.8 μg mL^− 1^-1.25 mg/mL^− 1^) of *P. lentiscus.* The tree provides a resinous exudate named mastic gum [62]. With reference to the biological behavior of oleanolic acid, it seems to beneficially modulate the peroxisome proliferator-activated receptors (PPAR) which are activated in several diseases e.g. diabetes mellitus, dyslipidemia and metabolic syndrome [63]. In a recent microbiological study, oleanolic acid synergized with the ß-lactam antibiotics ampicillin and oxacillin against the Gram-positive *S. aureus*, *S. epidermidis* and *Listeria monocytogenes* [42]. This can be attributed to the inhibition of the release of ß-lactamase, allowing for the easier eradication of methicillin-resistant *S. aureus* by ß-lactams [64]. Nevertheless, Shin et al. showed that oleanolic acid can act solely in synergy with aminoglycoside antibiotics such as kanamycin and cannot enhance the effectiveness of other antimicrobial agents e.g. tetracycline, norfloxacin and rifampicin against *Acinetobacter baumannii*. In that case the possible mechanism of action involves alteration in energy metabolism pathways and cell membrane susceptibility [65]. Another report underlined the superior inhibitory effects of oleanolic acid (MIC = 30 μg mL^− 1^-80 μg mL^− 1^ compared to several triterpene acids against oral streptococci and *E. faecalis* [66]. Interestingly, it seems that the interference of oleanolic acid with cell envelope, the structure and location of the acyl group on ring A results in a wide-spectrum antimicrobial action against Gram-positive and Gram-negative microorganisms [41, 42]. This was also confirmed in an earlier report on a novel pentacyclic triterpene, namely 3-oxo-olean-12(13),18(19)-dien-29α-carboxylic acid [67].

The natural tetracyclic triterpenoid 24Z-isomasticadienolic acid showed an enhanced inhibitory activity compared to oleanonic aldehyde against Gram-positive and Gram-positive anaerobic oral pathogens as well as streptococci. 24Z-isomasticadienolic acid has proven to possess anti-inflammatory traits against acute and chronic infections. In particular, 24Z-isomasticadienolic acid completely abolished the release of leukotriene B4 (LTB_4_) from polymorphonuclear leukocytes [68]. 24Z-isomasticadienolic (MBC = 0.2 mg/ml) and it exerted significant antibacterial effects against *Helicobacter pylori* strains (MBC = 0.35 mg/ml) [37]. Since there are no available data on the antimicrobial properties of oleanonic aldehyde, a pentacyclic triterpene, the current study contains the first promising results against oral bacteria.

## Conclusions

In conclusion, the present study highlighted the high-level antimicrobial efficacy of eight different constituents of *O. europaea* and *P. lentiscus* against a panel of nine different oral microorganisms. Overall, compounds from *O. europaea* and *P. lentiscus* such as maslinic acid and oleanolic acid were extremely effective against the tested oral pathogens, especially streptococci and anaerobic oral microorganisms. All tested extracts proved to be ineffective against the anerobic *P. intermedia*. Thus, future clinical studies should investigate the use of these natural antimicrobial agents in the treatment of caries- and periodontitis-related oral biofilms.

## Abbreviations

Avr: Avirulence
CBA: Columbia blood agar plates
CC: Column chromatography
CFU: Colony forming units
CH_2_Cl_2_: Dichloromethane
cHex: Cyclohexane
CHX: Chlorhexidine
CLSI: Clinical and Laboratory Standards Institute
DMSO: Dimethyl sulfoxide
*EfaA*: Endocarditis-related antigen A
EtOH: Ethanol
EVOO: Extra virgin olive oil
H_2_O: Water
HCB: Yeast-cysteine blood agar plates
HPLC: High performance liquid chromatography
IL: Interleukin
MBC: Minimum bactericidal concentration
MeOH: Methanol
MHB: Mueller-Hinton Broth
MIC: Minimum inhibitory concentration
MPLC: Medium pressure liquid chromatography
MS: Mass spectrometry
MW: Molecular weight
NMR: Nuclear magnetic resonance spectroscopy
NO: Nitric oxide
^−^OSCN: Hypothiocyanite
PPAR: Peroxisome proliferator-activated receptors
SDB: Sabouraud Dextrose Broth
Si: Silica
TLC: Thin layer chromatography
TMEWP: Total mastic extract without polymer
TNF-α: Tumor necrosis factor α
UV: Ultraviolet
WCB: Wilkins–Chalgren broth
